# Medicaid Accountable Care Model Designs and Maternal Health Measures

**DOI:** 10.1001/jamanetworkopen.2025.36565

**Published:** 2025-10-08

**Authors:** Megan B. Cole, Kenneth Lim, Kevin H. Nguyen, Sarah H. Gordon, Lois McCloskey, Collette Ncube, Elizabeth Patton, Amy Michals, Emily Sisson, Karen E. Lasser

**Affiliations:** 1 Department of Population Medicine, Harvard Medical School & Harvard Pilgrim Health Care Institute, Boston, Massachusetts; 2 Department of Health Law, Policy, and Management, Boston University School of Public Health, Boston, Massachusetts; 3 Department of Community Health Sciences, Boston University School of Public Health, Boston, Massachusetts; 4 Department of Epidemiology, Boston University School of Public Health, Boston, Massachusetts; 5 Department of Obstetrics & Gynecology, Boston Medical Center, Boston, Massachusetts; 6 Biostatistics and Epidemiology Data Analytics Center (BEDAC), Boston University School of Public Health, Boston, Massachusetts; 7 Division of General Internal Medicine, Boston Medical Center, Boston, Massachusetts

## Abstract

**Importance:**

There is significant heterogeneity in how Medicaid accountable care organizations (ACOs) are designed, but little is known about the association between different Medicaid ACO model designs and maternal health. In 2018, Massachusetts initiated a Medicaid ACO program with 2 distinct ACO models—model A (health system–managed care organization partnership) and model B (primary care practice [PCP]–led)—which may differentially affect coordination, integration, and quality for pregnant and postpartum patients.

**Objective:**

To estimate the differential association between Medicaid ACO model designs and maternal health care measures.

**Design, Setting, and Participants:**

This cohort study used the 2014 to 2020 Massachusetts All Payer Claims Database on Medicaid-covered deliveries among pregnant patients aged 18 years or older, indexed to the delivery-quarter. Data were analyzed September 2024 to March 2025.

**Exposure:**

Attribution to Medicaid ACO model A vs model B vs a non-ACO.

**Main Outcomes and Measures:**

Six quality of care–sensitive maternal health measures (severe maternal morbidity, preterm birth, cesarean delivery, timely postpartum care, postpartum depression screening, and postpartum glucose screening) and 2 health care utilization measures (office visits, emergency department [ED] visits) were measured during the prenatal period and at 60 days and 6 months post partum. Using a difference-in-differences (DID) approach, study measures were compared before (2016-2017) vs after (2018-2020) Medicaid ACO implementation among ACO model A vs model B vs non-ACO patients. Models adjusted for patient-level characteristics and included hospital, county, and delivery-month fixed effects.

**Results:**

Among 67 204 Medicaid-insured deliveries (mean [SD] maternal age, 28.1 [5.7] years), both ACO model A and model B were associated with increased probability of postpartum depression screening (DID for model A, 6.11 percentage points [pp]; 95% CI. 4.34-7.88 pp; DID for model B, 5.58 pp; 95% CI, 3.83-7.33 pp) compared with Medicaid non-ACO deliveries. Model A was associated with increased probability of a timely postpartum visit (DID, 5.18 pp, 95% CI, 4.34-7.88 pp) and decreased rates of prenatal ED visits (DID incidence rate ratio [IRR], 0.91, 95% CI, 0.84-0.98), whereas model B was associated with increased office visits during the prenatal and postpartum periods (eg, DID IRR for prenatal period, 1.10; 95% CI, 1.07-1.12). Changes in cesarean deliveries were inconclusive, with no other statistically significant changes in outcomes.

**Conclusions and Relevance:**

In this cohort study of Medicaid-insured deliveries, there was heterogeneity in the association between Medicaid ACOs and maternal health care measures by ACO model type. States should consider the differential implications of ACO design types and how to best optimize model designs to improve care and outcomes for pregnant and postpartum patients.

## Key Points

**Question:**

Are the 2 Medicaid accountable care organization (ACO) model designs implemented in Massachusetts differentially associated with process and outcome measures among pregnant and postpartum patients?

**Findings:**

This cohort study of 67 204 Medicaid-insured deliveries found that primary care practice–led ACOs were associated with increased perinatal office visits while health system–managed care organization partnership ACOs were associated with increased receipt of timely postpartum visits and decreased prenatal emergency department visits.

**Meaning:**

In this study, both Medicaid ACO models were associated with improved outcomes, but with some differences between models, suggesting that states should consider how to best optimize model designs to improve care and outcomes for pregnant and postpartum patients when designing or redesigning their programs.

## Teaser

This cohort study evaluates whether health and utilization outcomes for pregnant and postpartum patients in Massachusetts vary based on their enrollment in a health system–managed care organization partnership accountable care organization (ACO) vs a primary care practice–led ACO.

## Introduction

To promote integrated, coordinated, and value-based health care, accountable care organizations (ACOs) have evolved as the leading health care payment and delivery reform model in the United States.^1^ While there is heterogeneity in how ACOs are structured, a commonality of ACOs is that they bring together groups of health care entities, which may include primary care practices (PCPs), specialists, and/or hospitals, that are collectively responsible for cost and quality of care for their attributed patient population, with opportunity for shared savings if contractual cost and quality benchmarks are met. Early ACO models were primarily implemented within Medicare or privately insured populations,^2^ but Medicaid ACO participation has grown in recent years,^1^ with 13 states implementing Medicaid ACOs as of 2025.

There has also been growing recognition of a US maternal health crisis, particularly within Medicaid—the low-income health insurance program that covers 42% of US births.^3^ For instance, rates of severe maternal morbidity (SMM) have increased,^4^ especially within Medicaid; this includes Massachusetts, where SMM rates nearly doubled between 2011 and 2020.^3^ Meanwhile, prenatal and postpartum care visit rates remain inadequate among Medicaid enrollees, with subpar quality-of-care metrics and delivery outcomes.^5,6,7,8,9^ Medicaid ACOs, if effectively designed, could help improve maternal health care through maternal health–related pay-for-performance metrics, reductions in adverse pregnancy-related costs to achieve ACO shared savings, and systems-level integration and coordination of care within ACOs that may particularly benefit pregnant and postpartum patients, as they often interact with multiple clinicians across the preconception, perinatal, and postpartum periods.^10^

Evidence on the association between Medicaid ACOs and maternal health is limited but growing, with both promising and null findings.^11,12^ For example, our recent study reported Massachusetts’s Medicaid ACOs were associated with increased office visits during the prenatal and postpartum periods and some improvements in perinatal care process measures, but no changes in pregnancy-related outcomes.^13^ However, this study did not assess differences by ACO model design. Yet, this is important because there is heterogeneity in how Medicaid ACOs are designed, making it difficult to ascertain which Medicaid ACO model designs improve—or hinder—care. For instance, Medicaid ACOs vary in terms of their payment structure (eg, fee-for-service vs managed care) and level of specialist integration within the ACO.^14,15,16,17,18^ Evidence from Medicare ACOs suggests ACOs with more specialists and fewer primary care clinicians generally performed worse on disease prevention and wellness screening measures, but other studies report no consistent differences in quality by Medicare ACO type.^19,20^ However, little is known about the association between different Medicaid ACO model designs and care for Medicaid enrollees.

A natural experiment in Massachusetts offers opportunity to address this literature gap. In 2018, Massachusetts initiated a Medicaid ACO program that included 2 different ACO model types: (1) model A: a health system and Medicaid managed care organization (MCO) partnership model, where specialists (eg, obstetrician/gynecologists) are part of the ACO network, and (2) model B: a PCP-led model, where the ACO contracts with the state, and specialists are not directly part of the ACO. The objective of this study was to assess how these 2 Medicaid ACO model types were associated with utilization-related process (eg, office visits) and outcome measures (eg, cesarean deliveries) among pregnant and postpartum patients. We hypothesized that model A may be associated with larger magnitudes of improvement due to more opportunity for care coordination and integration and more direct financial incentives to minimize high-cost care.

## Methods

### Study Setting

In March 2018, the Massachusetts Medicaid program implemented an 1115 waiver to improve quality of care, coordination, and integration of care for Medicaid enrollees. Seventeen Medicaid ACOs formed across the state; each ACO included networks of PCPs, specialists, and hospitals responsible for health care costs and quality-of-care metrics (eTable 2 in Supplement 1) for attributed Medicaid members. Medicaid members were attributed to an ACO if their PCP was part of an ACO, and PCPs were only able to participate in a single Medicaid ACO; not all PCPs in the state participated (approximately 25% of Medicaid-serving practices did not participate in the ACO, including 1 large health system^21^). Fourteen Massachusetts Medicaid ACOs (covering approximately 50% of Medicaid ACO members) were implemented under the model A health system and MCO partnership model, where a health system (inclusive of hospitals, specialists, and PCPs) partnered with a single MCO to deliver all health care services for ACO members within the ACO network. In contrast, 3 Massachusetts ACOs (covering the remaining 50% of ACO members) were implemented under the model B primary care model, where groups of PCPs came together to form an ACO. In model B, primary care services were delivered by the ACO while other care was delivered through the state’s Medicaid fee-for-service network. Under both models, the ACOs were provider-led, shared savings were available with both upside and downside financial risk, and performance metrics were the same, although total cost-of-care benchmarks were calculated using different algorithms. eTable 1 in Supplement 1 provides further details on the ACO model types.

We followed the Strengthening the Reporting of Observational Studies in Epidemiology (STROBE) guidelines. The Boston University institutional review board approved the study with a waiver of informed consent because data were deidentified.

### Data Source and Study Population

Our primary data source was the 2014 to 2020 Massachusetts All Payer Claims Database, which includes complete claims and enrollment files for all Medicaid-insured and most privately insured persons in the state. Secondary data sources included the 2018 to 2020 Massachusetts Registration of Provider Organization files,^22^ which we used to link primary care practitioner national provider identifiers to ACOs; Massachusetts Medicaid provider directories, which included information on which practices belonged to which ACOs; and the American Community Survey data,^23^ from which we obtained zip code–area sociodemographic characteristics.

Our primary study population included Medicaid enrollees aged 18 years and older in Massachusetts who were attributed to a primary care practitioner and had evidence of a live hospital delivery from 2016 to 2020, with a minimum of 60 days of insurance enrollment before and after delivery. To attribute enrollees to a PCP and thus to one of the exposure or comparison groups, we used a utilization-based algorithm based on the PCP that provided the most historical primary care services, as described previously^13,24,25,26,27^ and in eTable 3 in Supplement 1. We further excluded deliveries that were dually enrolled in other insurance, occurring in the ACO transition period (quarters 1-2 of 2018), and with emergency-only Medicaid.

### Exposure Definition

Our study’s exposure was categorically defined: deliveries attributed to Medicaid ACO model A, to Medicaid ACO model B, and to non-ACO practices (comparison group). Assignment to 1 of these 3 groups was dependent on the ACO type in which an enrollee’s PCP participated, based on ACO participation status as of 2018; a PCP’s assignment to 1 of these groups was constant across the study period (2016-2020).

### Outcomes

We examined 2 sets of outcomes that may have been sensitive to changes in care delivery under the ACO and for which we could validly measure in our data. First, we examined 6 binary quality of care–sensitive maternal health measures, including 3 delivery-related outcomes and 3 process-oriented measures: SMM during delivery (as defined by the US Centers for Disease Control and Prevention,^28^ excluding blood transfusions and ventilation) (eTable 4 in Supplement 1), preterm birth (<37 weeks’ gestation), cesarean delivery, timely postpartum care visit (within 12 weeks of delivery), postpartum depression screening, and postpartum glucose screening among members with gestational diabetes. Of these, depression screening was the only measure included in the ACO pay-for-performance metrics. Timely prenatal care was included as an ACO performance metric but could not be validly assessed in our data. Second, we examined 2 measures of utilization—number of all-cause office visits and emergency department (ED) visits—during 3 separate time intervals of the perinatal period: prenatal, within 60 days post partum, and within 6 months post partum. Detailed outcome specifications are provided in eTable 5 in Supplement 1.

### Statistical Analysis

Delivery-quarter was the unit of analysis. We first descriptively compared characteristics of Medicaid ACO model A vs model B vs non-ACO enrollees. Next, we used a difference-in-differences (DID) approach to compare measures for Medicaid ACO model A vs model B vs non-ACO deliveries before (quarter 1 of 2016 to quarter 4 of 2017) vs after (quarter 3 of 2018 to quarter 4 of 2020) ACO implementation. Binary measures were examined using generalized linear models with a gaussian distribution and count measures were examined using a negative binomial distribution and log link. The interaction between the categorical exposure variable (ie, model A, model B, or non-ACO) and the postperiod indicator was our coefficient of interest, or the DID. All models included hospital, county, and delivery-month fixed effects and adjusted for patient-level confounders (age, presence of 9 separate clinical diagnoses, multiple gestation, parity number, number of insurance enrollment days during pregnancy) and select zip code–level characteristics (percentage of households under the poverty level and identifying as non-Hispanic Black, rurality), with robust standard errors clustered at the individual level to account for repeated deliveries. eTable 6 in Supplement 1 includes detailed covariate definitions. Two-tailed statistical tests were considered significant at α = .05. Data were analyzed September 2024 through March 2025 using Stata MP version 18.0 (StataCorp).

We conducted multiple robustness checks and sensitivity analyses. First, to test the parallel trends assumption, which is required for the DID estimates to be valid, we graphically and statistically examined preperiod linear trends in each outcome by exposure group (eTable 8 in Supplement 1). Second, given that our Medicaid non-ACO group was small in size, we reran all main analyses using privately insured deliveries as an alternative comparison group. Third, we excluded 2020 data from analyses given that COVID-19 may have differentially impacted our exposure groups.

## Results

Of the 67 204 unique deliveries in our study sample, observable characteristics of ACO-attributed model A (28 888 deliveries; mean [SD] maternal age, 28.1 [5.7] years) vs ACO-attributed model B (28 779 deliveries; mean [SD] maternal age, 28.3 [5.7] years) Medicaid-enrolled deliveries were largely similar (Table 1). When comparing Medicaid ACO vs Medicaid non-ACO enrollees, characteristics were similar, with several exceptions: for instance, on average, non-ACO enrollees resided in zip codes with fewer non-Hispanic Black residents (7.8% among non-ACO vs 15.0% among ACO), were more rural (1078 of 9537 [11.3%] vs 2422 of 57 667 [4.2%]), were less likely to receive their primary care from an FQHC (2632 [27.6%] vs 31 429 [54.5%]), and had higher prevalences of asthma (1631 [17.1%] vs 8016 [13.9%]), major depression (2117 [22.2%] vs 10 611 [18.4%]), and anxiety (2537 [26.6%] vs 11 476 [19.9%]), compared with ACO enrollees.

**Table 1.** Study Population Characteristics: Medicaid-Enrolled Deliveries Attributed to ACO Model A vs ACO Model B vs non-ACO, 2016-2020^a^

Characteristic	Deliveries, No. (%)			
	Comparison group, Medicaid non-ACO (n = 9537)	Treatment groups, Medicaid ACO		
		Both models (n = 57 667)	Model A, health system–MCO partnership (n = 28 888)	Model B, primary care practice–led (n = 28 779)
Maternal age, mean (SD), y	27.8 (5.7)	28.2 (5.7)	28.1 (5.7)	28.3 (5.7)
Insurance enrollment days in prenatal period, mean (SD)^b^	265.7 (28.7)	263.6(31.8)	263.1(32.6)	264.2 (30.0)
Parity, mean (SD), No.	1.4 (0.60)	1.4 (0.57)	1.3 (0.56)	1.4 (0.57)
Multiple gestation	191 (2.0)	980/ (1.7)	520 (1.8)	460 (1.6)
Clinical diagnoses at baseline^c^				
Diabetes	362 (3.8)	1845 (3.2)	924 (3.2)	892 (3.1)
Hypertension	677 (7.1)	4383 (7.6)	2311 (8.0)	2101 (7.3)
Hyperlipidemia	172 (1.8)	1096 (1.9)	578 (2.0)	547 (1.9)
Cardiovascular disease	124 (1.3)	807 (1.4)	404 (1.4)	432 (1.5)
Asthma	1631 (17.1)	8016 (13.9)	4304 (14.9)	3770 (13.1)
BMI 25-40	2213 (23.2)	15 397 (26.7)	7973 (27.6)	7428 (25.8)
BMI >40	801 (8.4)	5017 (8.7)	2687 (9.3)	2331 (8.1)
Major depression	2117 (22.2)	10 611 (18.4)	2218 (19.1)	5151 (17.9)
Anxiety (any)	2537 (26.6)	11 476 (19.9)	6211 (21.5)	5324 (18.5)
Primary care at FQHC, %	2632 (27.6)	31 429 (54.5)	15 773 (54.6)	15 627 (54.3)
Rural residence	1078 (11.3)	2422 (4.2)	1098 (3.8)	1353 (4.7)
Race and ethnicity of patient zip code, mean (SD), %				
Black non-Hispanic	7.8 (9.7)	15.0 (17.8)	14.1 (17.8)	15.9 (17.9)
Hispanic	17.2 (15.2)	23.9 (21.5)	24.6 (23.8)	23.2 (19.2)
White non-Hispanic	68.3 (21.1)	52.4 (26.5)	52.1 (27.0)	52.6 (26.1)
Income of patient zip code, mean (SD) households <FPL, %	11.7 (8.4)	13.7 (8.1)	14.2 (8.9)	13.2 (7.2)

Abbreviations: ACO, accountable care organization; BMI, body mass index (calculated as weight in kilograms divided by height in meters squared); FQHC, federally qualified health care; FPL, federal poverty level; MCO, managed care organization.

^a^ Characteristics represent the study population across the pooled study period.

^b^ Standard prenatal period includes the 280 days prior to delivery.

^c^ Clinical diagnoses selected based on prevalent chronic conditions that are known to be associated with pregnancy outcomes and utilization of health care services.

### Association Between ACO Model Type and Quality of Care–Sensitive Maternal Health Measures

As shown in Table 2, Medicaid ACO models A and B were both associated with reductions in probability of cesarean delivery (DID for model A, −4.00 percentage points [pp]; 95% CI, −6.49 to −1.52 pp; DID for model B, −3.28 pp; 95% CI, −5.72 to −0.84) (Figure) and increases in probability of postpartum depression screening (DID for model A, 6.11 pp, 95% CI, 4.34 to 7.88 pp; DID for model B, 5.58 pp; 95% CI, 3.83 to 7.33 pp) compared with the Medicaid non-ACO group. For both measures, the magnitude of effect was qualitatively similar for model A and model B. There was no significant change in outcomes for model A or model B when examining SMM, preterm birth, or postpartum glucose screening. However, we observed heterogeneity in receipt of a timely postpartum visit by ACO model type (Table 2 and Figure): compared with the Medicaid non-ACO group, model A was associated with a 5.18 pp increase (95% CI, 4.34-7.88 pp) in probability of a timely postpartum visit, whereas model B was associated with no change in probability of a timely postpartum visit.

**Table 2.** Association Between Medicaid ACO and Quality of Care–Sensitive Measures by ACO Model Type: DID Results^a^^,^^b^

ACO model	Mean (95% CI)		DID coefficient (95% CI), percentage points^d^	*P* value
	Preperiod (2016-2017)^c^	Postperiod (2018-2020)		
**SMM during delivery (per 10 000 deliveries)^e^**				
Non-ACO	26.6 (0.1-53.1)	53.0 (25.5-80.5)	0 [Reference]	NA
ACO model A	64.9 (46.7-83.1)	86.8 (68.0-105.5)	−0.04 (−0.49 to 0.41)	.87
ACO model B	52.3 (35.1-69.5)	91.9 (74.8-109.1)	0.31 (−0.16 to 0.78)	.20
**Cesarean delivery, %**				
Non-ACO	32.9 (31.1-34.6)	36.8 (35.1-38.5)	0 [Reference]	NA
ACO model A	32.2 (31.2-33.3)	32.2 (31.3-33.3)	−4.00 (−6.49 to −1.52)	.002
ACO model B	32.6 (31.6-33.6)	33.3 (32.4-34.1)	−3.28 (−5.72 to −0.84)	.008
**Preterm birth (<37 wk), %**				
Non-ACO	8.3 (7.2-9.4)	10.2 (9.2-11.2)	0 [Reference]	NA
ACO model A	7.2 (6.6-7.8)	8.6 (8.1-9.2)	−0.46 (−1.96 to 1.03)	.54
ACO model B	7.6 (7.1-8.2)	9.3 (8.7-9.8)	−0.24 (−1.72 to 1.24)	.75
**Timely postpartum visit,%^f^**				
Non-ACO	78.2 (76.6-79.7)	77.0 (75.5-78.4)	0 [Reference]	NA
ACO model A	79.0 (78.2-79.9)	83.0 (82.2-83.7)	5.18 (2.30 to 7.36)	<.001
ACO model B	79.7 (79.0-80.5)	79.4 (78.7-80.1)	0.91 (−1.25 to 3.06)	.41
**Postpartum depression screening, %^f^**				
Non-ACO	26.0 (24.5-27.4)	26.2 (24.9-27.5)	0 [Reference]	NA
ACO model A	21.8 (21.0-22.7)	25.9 (25.1-26.7)	6.11 (4.34 to 7.88)	<.001
ACO model B	17.0 (16.2-17.7)	22.0 (21.3-22.7)	5.58 (3.83 to 7.33)	<.001
**Postpartum glucose screening, %^f^**				
Non-ACO	13.3 (8.5-18.0)	16.2 (12.3-20.2)	0 [Reference]	NA
ACO model A	17.3 (12.8-21.9)	16.3 (13.8-18.9)	−3.99 (−11.11 to 3.13)	.27
ACO model B	18.1 (14.2-22.0)	19.8 (17.5-22.2)	−1.23 (−7.77 to 5.32)	.71

Abbreviations: ACO, accountable care organization; DID, difference-in-difference; NA, not applicable; SMM, severe maternal morbidity.

^a^ ACO model A is the health system–managed care organization partnership model type and model B is the primary care practice–led model type.

^b^ DID results are adjusted for age, a vector of diagnoses, number of insurance enrollment days during pregnancy, multiple gestation (if applicable), parity number, and patient zip code characteristics and include fixed effects for delivery month, county, and delivery hospital.

^c^ Preperiod and postperiod estimates are adjusted marginal effects.

^d^ Coefficients shown represent the DIDs, which are measured as the interaction between the categorical exposure variable (Medicaid ACO model A vs model B vs Medicaid non-ACO) and our postperiod indicator (quarter 1 of 2016 to quarter 4 of 2017 vs quarter 3 of 2018 to quarter 4 of 2020).

^e^ SMM excludes blood transfusion and ventilation.

^f^ Deliveries occurring in quarter 4 of 2020 were excluded from postpartum measures.

**Figure.**
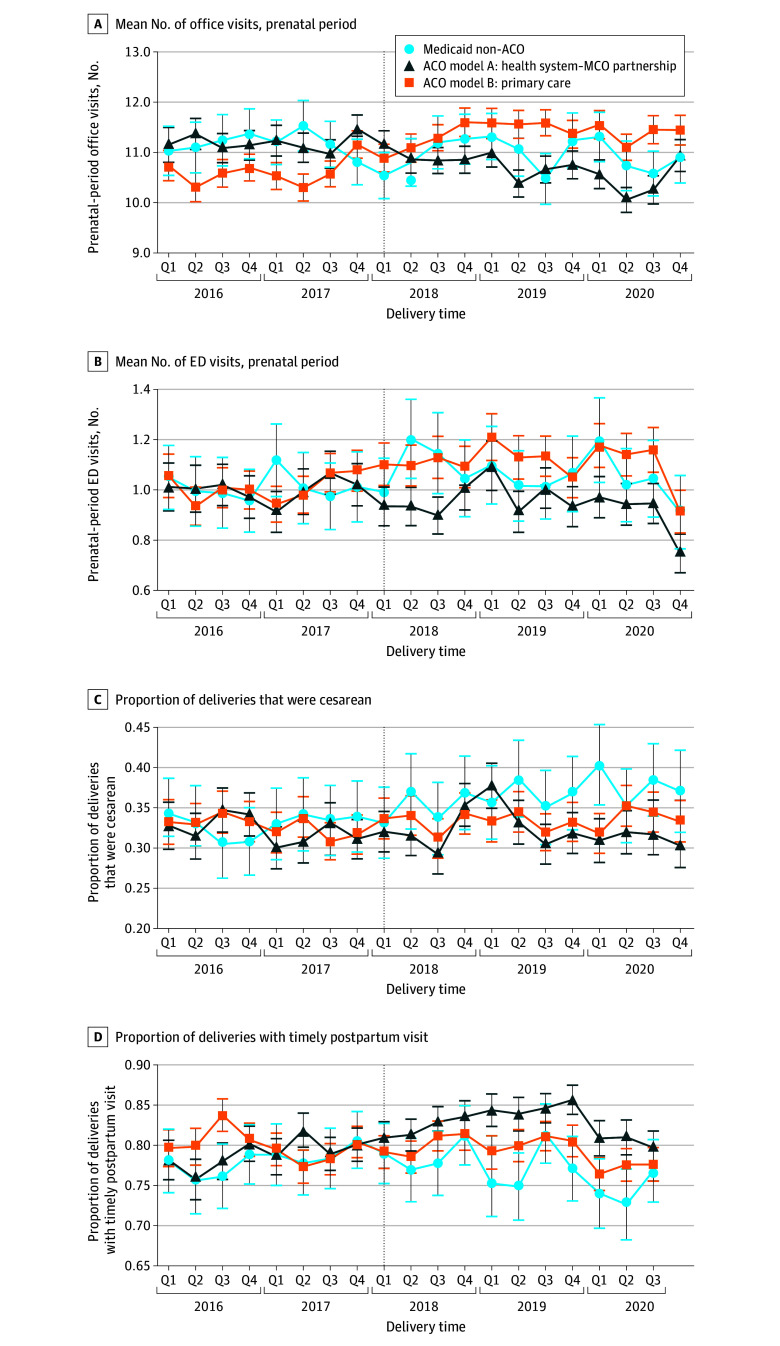
Trends in Select Study Outcomes by Medicaid Accountable Care Organization (ACO) Model Type, 2016-2020 Results are adjusted for age, a vector of diagnoses, number of insurance enrollment days during pregnancy, multiple gestation, parity number, and patient zip code characteristics and include fixed effects for delivery month, county, and delivery hospital. Coefficients shown represent the quarterly adjusted marginal effects. The vertical dotted line represents ACO implementation (effective quarter 2 of 2018). ED indicates emergency department; MCO, managed care organization.

### Association Between ACO Model Type and Health Care Utilization During the Perinatal Period

As shown in Table 3, Medicaid ACO model A was associated with a small decrease in number of all-cause office visits across the prenatal period and no statistical change in number of office visits at 60 days or 6 months post partum, while Medicaid ACO model B was consistently associated with increased rates of office visits across the perinatal period. For instance, compared with the Medicaid non-ACO group, model B was associated with an increase in number of office visits in the prenatal period (incident rate ratio [IRR], 1.10; 95% CI, 1.07-1.12; or 102 additional visits per 100 deliveries) (Figure), in the 60-day postpartum period (IRR, 1.16; 95% CI, 1.10-1.22; 7 additional visits per 100 deliveries), and in the 6-month postpartum period (IRR, 1.11; 95% CI, 1.06-1.16; 35 additional visits per 100 deliveries). Compared with the Medicaid non-ACO group, model A was associated with a decreased number of ED visits during the prenatal period (IRR, 0.91; 95% CI, 0.84-0.98; 10 fewer ED visits per 100 deliveries) (Figure). There were no other significant associations between either model type and ED visits.

**Table 3.** Association Between Medicaid ACO Model Type and Health Care Utilization During the Perinatal Period: DID Results^a^^,^^b^

ACO model	Preperiod (95% CI)^c^ (2016-2017)	Postperiod (95% CI)^c^ (2018-2020)	DID coefficient, IRR (95% CI)^d^	*P* value
**Office visits, prenatal period, mean No.**				
Non-ACO	11.18 (10.98-11.38)	11.00 (10.82-11.18)	1 [Reference]	NA
ACO model A	11.21 (11.09-11.32)	10.64 (10.54-10.74)	0.96 (0.94-0.99)	.004
ACO model B	10.62 (10.52-10.73)	11.46 (11.36-11.55)	1.10 (1.07-1.12)	<.001
**Office visits, 60 d post partum, mean No.^e^**				
Non-ACO	1.70 (1.64-1.76)	1.63 (1.58-1.69)	1 [Reference]	NA
ACO model A	1.70 (1.67-1.74)	1.68 (1.65-1.71)	1.03 (0.98-1.09)	.31
ACO model B	1.63 (1.60-1.66)	1.82 (1.78-1.85)	1.16 (1.10-1.22)	<.001
**Office visits, 6 mo postpartum, mean No.^e^**				
Non-ACO	3.49 (3.38-3.60)	3.18 (3.08-3.60)	1 [Reference]	NA
ACO model A	3.55 (3.49-3.61)	3.33 (3.27-3.38)	1.03 (0.98-1.08)	.24
ACO model B	3.43 (3.37-3.49)	3.47 (3.42-3.53)	1.11 (1.06-1.16)	<.001
**ED visits, prenatal period, mean No.**				
Non-ACO	1.01 (0.95-1.06)	1.06 (1.00-1.11)	1 [Reference]	NA
ACO model A	1.00 (0.96-1.03)	0.95 (0.92-0.97)	0.91 (0.84-0.98)	.02
ACO model B	1.01 (0.98-1.04)	1.12 (1.09-1.15)	1.06 (0.98-1.14)	.14
**ED visits, 60 d postpartum, mean No.^e^**				
Non-ACO	0.43 (0.39-0.46)	0.37 (0.34-0.41)	1 [Reference]	NA
ACO model A	0.40 (0.38-0.42)	0.35 (0.33-0.37)	0.98 (0.83-1.16)	.85
ACO model B	0.42 (0.40-0.44)	0.38 (0.36-0.40)	1.02 (0.87-1.20)	.83
**ED visits, 6 mo postpartum, mean No.^e^**				
Non-ACO	0.43 (0.34-0.41)	0.37 (0.34-0.41)	1 [Reference]	NA
ACO model A	0.40 (0.38-0.42)	0.35 (0.33-0.37)	0.99 (0.87-1.13)	.92
ACO model B	0.42 (0.40-0.44)	0.38 (0.36-0.40)	1.04 (0.92-1.17)	.57

Abbreviations: ACO, accountable care organization; DID, difference-in-difference; ED, emergency department; IRR, incident rate ratio; NA, not applicable.

^a^ ACO model A is the health system–managed care organization partnership model type and model B is the primary care practice–led model type.

^b^ DID results are adjusted for age, a vector of diagnoses, number of insurance enrollment days during pregnancy, multiple gestation, parity number, and patient zip code characteristics and include fixed effects for delivery month, county, and delivery hospital.

^c^ Preperiod and postperiod estimates are adjusted marginal effects.

^d^ Coefficients shown represent the DIDs, which are measured as the interaction between the categorical exposure variable (Medicaid ACO model A vs model B vs Medicaid non-ACO) and postperiod indicator (quarter 1 of 2016 to quarter 4 of 2017 vs quarter 3 of 2018 to quarter 4 of 2020). All DID coefficients are reported as IRRs, where an IRR greater than 1.0 indicates a positive association between the Medicaid ACO and the outcome.

^e^ Deliveries occurring in quarter 4 of 2020 were excluded from 60-day postpartum measures, and deliveries occurring in quarters 3 and 4 of 2020 were excluded from 6-month postpartum measures.

### Sensitivity Analyses and Robustness Checks

We observed no statistically significant differences in preperiod trends for our Medicaid ACO vs non-ACO groups. Further sensitivity analyses were largely consistent with our main findings, with 2 exceptions. First, when using privately insured individuals as an alternative control group, we observed no association between the Medicaid ACO and cesarean delivery rates for both model A and model B ACOs (eTable 7 in Supplement 1). Second, when using privately insured individuals as an alternative control group, we observed an association between the Medical ACO and probability of a timely postpartum visit for both model A and model B ACOs; however, the magnitude of association was larger for model A vs model B. eTables 7 to 9 in Supplement 1 present complete sensitivity results, including expanded discussion of preperiod trends.

## Discussion

During the first 3 years of Medicaid ACO implementation in Massachusetts, the Medicaid ACO was associated with relative increases in prenatal and postpartum office visits and some relative improvements in process-oriented quality of care–sensitive measures (ie, depression screening, timely postpartum care), with no change in other study outcomes. The health system–MCO partnership ACO (model A) design vs the PCP-led ACO (model B) design were differentially associated with some, but not all, outcomes. Observed increases in timely postpartum care were observed in the health system–MCO partnership model A design, whereas increases in office visits during the prenatal and postpartum periods were observed in the PCP-led ACO model B design. Furthermore, model A was associated with decreases in ED visits during the prenatal period. For all other study outcomes, results were consistent by ACO model type.

Both Medicaid ACO model types were associated with improvements in care for pregnant and postpartum patients, with some differences between the 2 model types. The PCP-led ACO model B was associated with increases in office visits in the prenatal and postpartum periods, while the health system–MCO partnership model A was not. This finding may be due to several factors. First, this may be due to differences in choice of PCPs, specialists, and hospitals, where model B members can see any maternity clinician that accepts Medicaid, whereas model A members are encouraged to see a maternity clinician within their ACO network. This could result in model B members being more likely to select a maternity clinician within proximity, who has more appointment availability, or with whom they have an existing relationship, thus increasing engagement rates. Alternatively, model B ACOs may be better positioned to initially engage new members who qualify for the ACO based on pregnancy status, but this requires further qualitative study. Moreover, it is possible that some of the increases in office visits within model B were not clinically advantageous, whereas model A may have improved optimization of beneficial health care utilization; although, increased visit rates also present increased opportunity for addressing medical, behavioral, and social service needs.

Despite model A ACOs generally not being associated with changes in frequency of care engagement in the postpartum period, they were associated with increased probability of a timely postpartum visit, unlike model B. This may be due to the fact the model A PCPs, specialists, and hospitals are more integrated (ie, part of the same health system and MCO network); thus, the initial postpartum linkage may be easier to facilitate when electronic health records or appointment systems are shared between a delivery hospital and maternity care clinician. It could also be because timely postpartum care is a metric incorporated into National Committee for Quality Assurance MCO star ratings,^29^ although the metric was not included within Massachusetts’ MCO quality reporting as of 2020.^30^

This study adds to the growing literature on the association between Medicaid ACOs and quality of care for pregnant and postpartum people and is, to our knowledge, the first study to assess how different Medicaid ACO model types may be differentially associated with health care measures. Our study found that Medicaid model B ACOs were associated with increases in all-cause office visits in the prenatal period, while findings from Oregon’s ACO-like Medicaid coordinated care organizations (CCOs)^12^ showed no change in number of prenatal visits—different but correlated measures.^12^ Of note, our study found improved prenatal engagement in PCP-led ACOs but not health system–MCO partnership-led ACOs, the latter of which share more similarities with the CCO model design; although, unlike Massachusetts’s Medicaid ACOs, Oregon CCOs are regional and operate under a global budget.^31^ Our findings are consistent with those of Henke et al,^11^ where hospital discharge data suggested some reductions in cesarean delivery rates and no change in SMM rates following Medicaid ACO implementation in Oregon and Colorado. Colorado’s Medicaid ACO program was implemented through regional accountable organizations that are led by PCPs, which more closely mirror Massachusetts’s model B ACOs. However, none of these studies were able to directly compare Medicaid ACO model types. While findings from the Medicare ACO literature suggest that different ACO models perform relatively similarly on quality measures,^20^ with some evidence that physician-led ACOs (but not hospital-integrated ACOs) are associated with ACO cost savings,^32,33^ we add to the literature by focusing on Medicaid ACO designs. We do so by using a novel nested natural experiment that included 2 distinct ACO model types, using a range of perinatal health care measures and a longer period of ACO implementation time compared with most Medicaid ACO studies.

Findings from this study have 3 key policy implications. First, PCP-led Medicaid ACO models may hold promise for increasing engagement in care among pregnant and postpartum people. This is important given that rates of prenatal and postpartum care engagement are often inadequate in Medicaid populations,^34^ and increasing the frequency of care engagement could result in better longer-term health outcomes. However, more research is needed to understand whether these are clinically beneficial visits. Second, health system–MCO partnership ACOs may be more promising for other types of perinatal outcomes, including increasing timely postpartum care and reducing ED use during the prenatal period. For both model types, more research is needed to identify the specific aspects of the model design that may have supported improved care use or quality. Third, more broadly, it is important that states consider how different model designs may differentially affect pregnant patients—and other types of clinical populations—when implementing or reforming Medicaid ACO programs. This is important for the 37 states without Medicaid ACOs, if they consider implementation, and for the 13 states with active Medicaid ACOs that may consider redesigns over time. This may be considered within the broader context of ACO model feasibility, where health system–MCO partnership models require a great degree of health systems integration to implement, whereas primary care models may be more feasible to implement in less integrated environments or states.

### Limitations

Our study has limitations. First, there is provider-level selection into Medicaid ACOs and into ACO model types, which may bias results. While our analytic approach aims to minimize the influence of this bias, residual confounding may exist. Second, our study sample excluded 24% of deliveries that were unattributed to a PCP, and thus our findings may not generalize to individuals unengaged in care. Third, our study was unable to subclassify office visits; we were unable to differentiate between appropriate vs avoidable visits. Additionally, claims data may underreport some diagnoses and services, especially services covered by maternity global billing. This may underestimate the overall rate for some study measures (eg, postpartum depression screening), while it also limits our ability to validly identify what occurs during perinatal visits. Furthermore, our study does not include measures that contextualize value (eg, changes in total cost of care), which should be the focus of future work.

## Conclusions

In this cohort study of Medicaid-enrolled deliveries, Medicaid ACOs were associated with increases in office visits during the prenatal and postpartum periods and improvements in some care process measures, but for some of these outcomes, there was heterogeneity by ACO model design. In designing or redesigning Medicaid ACO programs, states should consider the differential impact of different ACO designs and how to best optimize model designs to improve care and outcomes for pregnant and postpartum patients.

## Notes

Supplement 1. **eTable 1.** A Comparison of Key ACO Design Characteristics by Model Type **eTable 2.** Complete List of Massachusetts Medicaid ACO Performance Measures (2020) **eTable 3.** Qualifying Primary Care Visits **eTable 4.** Severe Maternal Morbidity Per 10000 Deliveries by Individual Indicator **eTable 5.** Study Measure Definitions **eTable 6.** Study Covariate Definitions **eTable 7.** Association Between Medicaid ACO Model Type and Outcomes When Using Privately Insured Individuals as the Control Group **eTable 8.** Preperiod Trends for Medicaid ACO vs Medicaid non-ACO Deliveries, 2016-2017 **eTable 9.** Difference-in-Differences Model Type Results Excluding 2020 Data

Supplement 2.
Data Sharing Statement
