# Supplementary material for: Medicaid Accountable Care Model Designs and Maternal Health Measures
Source: JAMA Netw Open. 2025 Oct 8;8(10):e2536565. doi: 10.1001/jamanetworkopen.2025.36565 (PMC12509007; doi:10.1001/jamanetworkopen.2025.36565)
Supplement: Supplement 1. — eTable 1. A Comparison of Key ACO Design Characteristics by Model Type eTable 2. Complete List of Massachusetts Medicaid ACO Performance Measures (2020) eTable 3. Qualifying Primary Care Visits eTable 4. Severe Maternal Morbidity Per 10000 Deliveries by Individual Indicator eTable 5. Study Measure Definitions eTable 6. Study Covariate Definitions eTable 7. Association Between Medicaid ACO Model Type and Outcomes When Using Privately Insured Individuals as the Control Group eTable 8. Preperiod Trends for Medicaid ACO vs Medicaid non-ACO Deliveries, 2016-2017 eTable 9. Difference-in-Differences Model Type Results Excluding 2020 Data [file jamanetwopen-e2536565-s001.pdf]

Supplemental Online Content

Cole MB, Lim K, Nguyen KH, et al. Medicaid accountable care model designs and maternal health measures. *JAMA Netw Open*. 2025;8(10):e2536565. doi:10.1001/jamanetworkopen.2025.36565

eTable 1. A Comparison of Key ACO Design Characteristics by Model Type

eTable 2. Complete List of Massachusetts Medicaid ACO Performance Measures (2020)

eTable 3. Qualifying Primary Care Visits

eTable 4. Severe Maternal Morbidity Per 10000 Deliveries by Individual Indicator

eTable 5. Study Measure Definitions

eTable 6. Study Covariate Definitions

eTable 7. Association Between Medicaid ACO Model Type and Outcomes When Using Privately Insured Individuals as the Control Group

eTable 8. Preperiod Trends for Medicaid ACO vs Medicaid non-ACO Deliveries, 2016-2017

eTable 9. Difference-in-Differences Model Type Results Excluding 2020 Data

This supplemental material has been provided by the authors to give readers additional information about their work.

Description of Massachusetts Medicaid ACOs

eTable 1. A Comparison of Key ACO Design Characteristics by Model Type

	ACO Model A (Health System and MCO Partnership Model)	ACO Model B (Primary Care Model)
**Organizational structure**	Provider-led; Made up of a health system – inclusive of primary care practices, specialists, and hospital(s) – that partners with a single Medicaid managed care organization	Provider-led; Made up of primary care practices that directly contract with state Medicaid program
**Network and service delivery structure**	Services are delivered within the ACO's health system/MCO network; enrollees use this ACO/MCO network for all medical (e.g., primary care, OB/GYN) care	Primary care services are delivered by the ACO; enrollees use the state Medicaid program network for all other medical (e.g., OB/GYN) care
**Payment model (payments to providers)**	Risk-adjusted, capitated managed care payments (prospective)	Fee for service payments (retrospective)
**Risk structure**	Shared savings with upside and downside risk; 100% insurance risk, with risk corridors	Shared savings with upside and downside risk; performance risk (not insurance risk), with risk corridors
**Performance metrics**	Report on, and receive financial incentives for, 18-20 metrics depending on the year (metrics are the same across model types), ACO is accountable for achieving quality targets to realize full shared savings.	Report on, and receive financial incentives for, 18-20 metrics depending on the year (metrics are the same across model types). ACO is accountable for achieving quality targets to realize full shared savings.
**Total cost of care (TCOC) accountability**	Accountable under a prospective payment system (which reflects both utilization and provider negotiated rates), including medical, behavioral health and pharmacy spend.	Accountable for a price-normalized TCOC Benchmark (which reflects utilization but not prices), including medical, behavioral health and pharmacy spend.

© 2025 Cole MB et al. *JAMA Network Open*.

Description of Massachusetts Medicaid ACO performance measures

eTable 2. Complete List of Massachusetts Medicaid ACO Performance Measures (2020)

Measure name	Description
1. Childhood Immunization Status	The percentage of children 2 years of age who had the following vaccines by their second birthday:<br>• Four diphtheria, tetanus and acellular pertussis (DTaP)<br>• Three polio (IPV); one measles, mumps, and rubella (MMR)<br>• Three H influenza type B (HiB); three hepatitis B (HepB)<br>• One chicken pox (VZV); four pneumococcal conjugate (PCV)<br>• One hepatitis A (HepA); two or three rotavirus (RV)<br>• Two influenza (flu) vaccines by their second birthday.
2. Adolescent Immunization Status	The percentage of adolescents 13 years of age who had one dose of meningococcal conjugate vaccine, one tetanus, diphtheria toxoids and acellular pertussis (Tdap) vaccine, and have completed the human papillomavirus (HPV) vaccine series by their 13th birthday. This measure calculates a rate for each vaccine and two combination rates.
3. Timeliness of Prenatal Care	The percentage of deliveries that were preceded by a prenatal care visit within the first trimester, on the attribution start date or within 42 days of ACO attribution
4. Asthma Medication Ratio	The percentage of ACO attributed members 5 to 64 years of age with persistent asthma and had a ratio of controller medications to total asthma medications of 0.50 or greater during the measurement year
5. Metabolic Monitoring for Children and Adolescents on Antipsychotics	The percentage of members 1 to 17 years of age who had two or more antipsychotic prescriptions and had metabolic testing
6. Comprehensive Diabetes Care – HbA1c Poor Control	The percentage of ACO attributed members 18 to 64 years of age with diabetes (type 1 and type 2) who had HbA1c poor control (>9.0%). Excludes members with a diagnosis of gestational diabetes.
7. Controlling High Blood Pressure	The percentage of ACO attributed members ages 18-64 who had a diagnosis of hypertension (HTN) and whose BP was adequately controlled (<140/90 mm Hg). Excludes pregnancy.
8. Hospital Readmissions (Adult)	For members ages 18 to 64, the number of acute inpatient stays during the measurement year that were followed by an acute unplanned readmission for any diagnosis within 30 days, compared to the predicted probability of an acute readmission. Excludes stays with principal diagnosis of pregnancy, or a condition originating in perinatal period
9. Acute Unplanned Admissions for Individuals with Diabetes	Case-mix adjusted rate of acute unplanned hospital admissions for individuals 18 to 64 years of age with diabetes
10. ED Visits for Adults with SMI or SUD	The risk adjusted ratio of the number of observed ED visits to the expected number of ED visits for ACO attributed members, ages 18 to 64, identified with a diagnosis of serious mental illness and/or substance use disorder
11. Follow-up after Hospitalization for Mental Illness (7 days)	The percentage of discharges for members 6 to 64 years of age who were hospitalized for treatment of selected mental illness

© 2025 Cole MB et al. *JAMA Network Open*.

	diagnosis and who received a follow-up visit with a mental health practitioner within 7 days of discharge
12. Follow-up after ED visit for Mental Illness (7 days)	The percentage of emergency department (ED) visits for members ages 6 to 64 with a principal diagnosis of mental illness or intentional self-harm, who had a follow-up visit for mental illness within 7 days of the ED visit.
13. Depression Screening and Follow-up Plan	The percentage of ACO attributed members ages 12 to 64 with an outpatient visit during the measurement year who are screened for clinical depression using a standardized tool during the measurement year AND, if screened positive, have a follow-up plan documented on the date of the positive screen
14. Depression Remission or Response	The percentage of members ages 12 to 64 with a diagnosis of depression and an elevated PHQ-9 score who received follow-up testing with PHQ-9 and had evidence of remission or response between 4 and 8 months of the elevated score
15. Initiation and Engagement of Alcohol, Opioid or Other Drug Abuse or Dependence Treatment	The percentage of ACO members ages 13 to 64 with a new episode of alcohol, opioid, or other drug (AOD) abuse or dependence who received the following:<br>Initiation: An inpatient AOD admission, outpatient visit, intensive outpatient encounter or partial hospitalization, telehealth, or medication assisted treatment (MAT) within 14 days of the diagnosis<br>Engagement: Two or more additional AOD services or MAT within 34 days of the initiation visit
16. Oral Health Evaluation	The percentage of ACO attributed members under age 21 who received a comprehensive or periodic oral evaluation as a dental service in the measurement year
17. Health-Related Social Needs Screening	The percentage of ACO attributed members ages 0 to 64 who were screened for health-related social needs in the measurement year. All topics must be on the survey, and patient needs to answer all core and at least 1 supplemental topic to qualify<br><br>Core domains for screening (must answer all):<br>•Food<br>•Housing<br>•Transportation<br>•Utility<br><br>Supplemental domains (must answer at least 1):<br>•Employment, training or education<br>•Experience of violence<br>•Social supports
18. Community Tenure	Percentage of eligible days that ACO members, ages 0 –64, with BH diagnoses and/or at least 3 consecutive months of LTSS utilization, reside in their home or a community setting without utilizing acute, chronic or post-acute institutional health care services during the measurement year
19. LTSS Community Partner Engagement	Percentage of assignments to LTSS community partners among ACO attributed members ages 3 to 64 with documentation of engagement within 122 days of each assignment
20. Behavioral Health Community Partner Engagement	Percentage of assignments to Behavioral Health Community Partners among ACO-attributed members ages 18 to 64 with documentation of engagement within 122 days of each assignment

© 2025 Cole MB et al. *JAMA Network Open*.

Primary care provider (PCP) and ACO attribution methodology

In order to assess whether or not a delivery was assigned to the ACO versus non-ACO group, we used a utilization-based approach based on the member's utilized PCP, as ACO assignment was dependent on a member's PCP (i.e., every PCP in Massachusetts either did or did not participate in the Medicaid ACO, and each PCP could only participate in a single Medicaid ACO). For deliveries occurring prior to implementation of the ACO (i.e., in 2016-2017), we classified deliveries as in the "ACO treatment group" (i.e., eventual ACO group) if the PCP participated in the ACO as of 2018.

We first attributed each delivery to a primary care provider (PCP), or primary care practice. To do so, we first attributed each delivery to the PCP with whom the member had the most recent preventive or well evaluation and management (E&M) visit (Exhibit A2, level 1), by examining claims billed during the 24 months prior to delivery. If there were no preventive or well E&M visits with a PCP, then we assigned the delivery to the PCP with whom they had the most recent "other E&M visit," inclusive of sick visits (Exhibit A2, level 2). If no level 1 or level 2 visits took place with a PCP in the 24 months prior to delivery, then we assigned the delivery to the PCP with whom they had the most recent "other preventive service" (Exhibit A2, level 3). Deliveries with no evidence of any primary care use in the 24 months prior to delivery were unattributed and thus excluded from our sample, as we had no way of assigning them to the ACO vs non-ACO group.

After assigning each delivery to a PCP, we linked these PCP assignments with PCP directories that captured whether or not each PCP was in an ACO or not in an ACO, which determined treatment assignment. Our study team created these PCP to ACO crosswalk directories based upon the Massachusetts RPO, Medicaid provider directories, and publicly available lists of primary care practices within each Medicaid ACO. If a member's assigned PCP (i.e. their NPI) was not in our directory, then the member was excluded from our sample, as we had no way to assign them to the intervention vs comparison group.

eTable 3. Qualifying Primary Care Visits

**Level 1: Preventive or well E&M visits with a PCP**
ICD-9: V70.0*, V20. 2*<br>ICD-10: Z00.0*, Z00.12*<br>CPT/HCPCS: 99381–99397, G0438, G0439, G0402, G0513, G0514
**Level 2: Other E&M visits with a PCP**
Other E&M or sick visit:<br>CPT/HCPCS: 99201, 99202, 99203, 99204, 99205, 99211, 99212, 99213, 99214, 99215, 99242-99245, T1015<br>Other care management:<br>CPT/HCPCS: G0506, G0511, 99424- 99427, 99487-99491, 99490-99491, G0108, G0109, G0270, G0271
**Level 3: Receipt of other preventive services with a PCP**
ICD-9/ICD-10/CPT/HCPCS codes associated with following services:<br>Glucose test; lipid test; tobacco screening, education, or counseling; depression or behavioral health screening; weight, diet, or nutrition counseling; flu vaccine; other vaccines; hearing test or audiology screening; developmental screening or testing; other counseling for behavioral change; other health status or risk screening

© 2025 Cole MB et al. *JAMA Network Open*.

Outcome specifications

Severe Maternal Morbidity (SMM) measure

While the Centers for Disease Control and Prevention (CDC) includes 21 clinical morbidity indicators in their measure of SMM as of 2023, our main analyses excluded 2 of these 21 indicators: blood transfusions and ventilation. We excluded blood transfusions based on literature suggesting that inclusion of blood transfusions in SMM may not be valid, as it overestimates morbidity by flagging minor transfusions. We excluded ventilation after observing elevated rates of ventilation in our data – we hypothesized that this may be due to infant ventilation being billed on the mother's claims during the hospital stay. Even after excluding all claim lines with diagnoses of newborn respiratory distress, our ventilation rates remained elevated above what seemed reasonable. Thus, we excluded ventilation from our SMM measure. However, we included both blood transfusions and ventilation in SMM when conducting sensitivity analyses. Rates of SMM by individual indicator are shown below (eTable 4), inclusive of blood transfusions and ventilation.

eTable 4. Severe Maternal Morbidity Per 10000 Deliveries by Individual Indicator

SMM indicator	Number per 10,000 deliveries (2016-2020)
1. Acute myocardial infarction	1.19
2. Aneurysm	0.93
3. Acute renal failure	23.39
4. Adult respiratory distress syndrome	14.32
5. Amniotic fluid embolism	0.76
6. Cardiac arrest/ventricular fibrillation	1.44
7. Conversion of cardiac rhythm	0.42
8. Disseminated intravascular coagulation	25.34
9. Eclampsia	16.52
10. Heart failure/arrest during surgery or procedure	0.08
11. Puerperal cerebrovascular disorders	8.22
12. Pulmonary edema / Acute heart failure	12.54
13. Severe anesthesia complications	0.85
14. Sepsis	13.90
15. Shock	12.20
16. Sickle cell disease with crisis	1.36
17. Air and thrombotic embolism	5.59
18. Blood products transfusion	132.19
19. Hysterectomy	2.12
20. Temporary tracheostomy	0.25
21.Ventilation	81.10
SMM (any evidence of 21 indicators)	298.54
SMM (excluding blood trans and ventilation)	106.94

© 2025 Cole MB et al. *JAMA Network Open*.

Other study measure definitions

The measures examined in our study are listed below (eTable 5), each with details on how they were defined and operationalized. We also examined number of unique inpatient admissions (all cause) during the prenatal and postpartum periods. However, we do not include these analyses in our paper, as lack of parallel pre-period trends prevented evaluation.

eTable 5. Study Measure Definitions

Study measure	Definition or codes
Cesarean section (versus vaginal) delivery	Presence of one of the following codes on claims billed during delivery hospitalization:<br><br>ICD10: O82.X, O84.2, Z38.01, Z38.31, Z38.62, Z38.64, Z38.66<br>CPT: 59510, 59514, 59515, 59618-59622
Preterm birth	Delivery prior to 37 weeks gestation, where number of weeks gestation at time of delivery was calculated using Z3A* ICD10 codes billed during delivery. If no Z3A codes were present during delivery, then we flagged any Z3A code billed during pregnancy and used the code billed closest to the date of delivery to then calculate weeks gestation at time of delivery (e.g., if Z3A36, which indicates 36 weeks gestation, was billed for a service received 2 weeks prior to delivery, then weeks gestation at time of delivery would be 36+2=38 weeks).
Severe maternal morbidity (SMM)	Binary indicator based on presence of at least 1 of 19 morbidity indicators included on claims billed during the delivery hospitalization window. See Table S4.
Timely postpartum visit	Receipt of a postpartum visit within 60 days of delivery discharge. This includes any comprehensive postpartum office visit with a maternity care provider (e.g., OB/GYN, midwife, family practitioner PCP).<br><br>We used 60 days (versus 12 weeks, or 84 days) due to the fact that some postpartum people lose Medicaid coverage after 60 days and may thereafter become unobservable.
Postpartum depression screening	Receipt of a depression screening or behavioral health assessment within 60 days postpartum:<br>ICD10: Z13.32, Z13.89<br>CPT: 99420, 96127, 96110, 96160, 96161, 96146, S3005, G0444
Postpartum glucose screening	Among deliveries with any prenatal diagnosis of diabetes or gestational diabetes, receipt of a glucose tolerance test within 60 days of delivery discharge:<br>ICD10: O99.815, O24.439<br>CPT: 82947, 82948, 82950, 82951, 82952
Number of office visits: prenatal period; postpartum period (30 days, 60 days, 6 months)	Count of unique office visits (all cause) during: (1) the prenatal period (between 4 weeks gestation and delivery date); (2) 30 days postpartum; (3) 60 days postpartum; (4) 6 months (180 days) postpartum. Office visits included those billed as outpatient facility claims (excluding ED visits) and professional claims.
Number of emergency department visits: prenatal period; postpartum period (30 days, 60 days, 6 months)	Count of unique emergency department visits (all cause), excluding those that resulted in inpatient admission, during: (1) the prenatal period (between 4 weeks gestation and

© 2025 Cole MB et al. *JAMA Network Open*.

	delivery date); (2) 30 days postpartum; (3) 60 days postpartum; (4) 6 months (180 days) postpartum.<br>Revenue code (REV): 0450,0451,0452, 0453, 0454, 0455, 0456, 0457, 0458, 0459, 0981<br>Place of service (POS): 23

© 2025 Cole MB et al. *JAMA Network Open*.

Other covariates definitions

The covariates examined in our study are listed below (eTable 6), each with details on how they were defined and operationalized.

eTable 6. Study Covariate Definitions

Study covariate	Definition
Age	Age at time of delivery, as reported on the medical claims (##.#)
Clinical diagnoses	A member was classified as having a clinical diagnosis based on ICD-9 and ICD-10 codes that were billed in the 24 months prior to delivery. To be classified as having a diagnosis, one inpatient claim or two or more other claim types were required to include the diagnosis. The following clinical diagnoses were included: diabetes, hypertension, hyperlipidemia, cardiovascular disease, asthma, BMI 25-40, BMI >40, major depression, other depression, anxiety
Insured days	This is the number of insurance enrollment days in the 280 days (40 weeks) prior to delivery, also known as the prenatal period. Of note, for many deliveries (e.g., deliveries occurring before 40 weeks gestation; enrollment in Medicaid during the first trimester), a member may be enrolled for the duration of their known pregnancy, but have fewer than 280 days of enrollment.
Multiple gestation	Multiple gestation (yes/no) was defined as having a delivery with one of the following multiple gestation codes billed during the inpatient hospital stay:<br>ICD-10: O30.*, O84.*, Z37.2, Z37.3, Z37.5*, Z37.6*, Z38.3-Z28.8<br>CPT: 59400, 59510-22, 59510, 59400, 59510-22, 59510
Parity number	Number of live deliveries (1-*n)* captured in the dataset, as of delivery *d*. For example, if a member gives birth in December 2015 and in March 2017, the December 2015 delivery would have a parity value of 1 and the March 2017 delivery would have a parity value of 2.
Rural residential zip code	Using the member's 5 digit residential zip code at time of delivery, we classified a member as living in a rural area (yes/no) if the zip code met the definition established by the Massachusetts Office of Rural Health (including level 1 and level 2 rural zip codes), where a municipality in Massachusetts is considered rural if it meets one of the following criteria: (1) meets at least one of three federal rural definitions at the sub-county level (Census Bureau, OMB, or. RUCAs), and/or (2) has a population less than 10,000 people and a population density below 500 people per square.
Other patient zip code characteristics	Racial/ethnic composition of patient zip code (e.g., percent Black non-Hispanic), percent of households below the federal poverty level, and median household income were all derived from the 2018 American Community Survey (ACS) data.
Delivery hospital	We identified the universe of facility NPIs for acute hospitals in Massachusetts that had labor and delivery services at any point in the 2016-2020 study period. Delivery hospital (categorical, with 45 unique values) was based on the facility NPI (billing provider) included on the inpatient hospital claims billed during the delivery stay. If a delivery had >1 hospital NPI billed during the delivery window (2.0% of deliveries), we assigned the delivery to the most frequently billed hospital.

© 2025 Cole MB et al. *JAMA Network Open*.

Sensitivity analyses and robustness checks

eTable 7. Association Between Medicaid ACO Model Type and Outcomes When Using Privately Insured Individuals as the Control Group

eTable 7-A. Association between Medicaid ACO model type[a] and maternal health care measures when using an alternative, privately insured control group: difference-in-differences results[b] (2016-2020)

	Coefficient (DID)[c]	95% CI		P-value
SMM during delivery[d] (per 10,000 deliveries)				
non-ACO (private)	ref	ref	ref	ref
ACO Model A	-10.46	-38.61	17.69	0.466
ACO Model B	8.75	-18.17	35.67	0.524
Cesarean section delivery, %				
non-ACO (private)	ref	ref	ref	ref
ACO Model A	-0.38	-1.93	1.16	0.625
ACO Model B	0.28	-1.21	1.77	0.716
Pre-term birth (<37 weeks), %				
non-ACO (private)	ref	ref	ref	ref
ACO Model A	-0.72	-1.61	0.16	0.110
ACO Model B	-0.54	-1.40	0.31	0.212
Timely postpartum visit[e], %				
non-ACO (private)	ref	ref	ref	ref
ACO Model A	9.45	8.11	10.79	<0.001
ACO Model B	5.20	3.89	6.50	<0.001
Postpartum depression screening[e], %				
non-ACO (private)	ref	ref	ref	ref
ACO Model A	2.32	0.19	4.44	0.032
ACO Model B	3.16	1.08	5.24	0.003
Postpartum glucose screening[e], %				
non-ACO (private)	ref	ref	ref	ref
ACO Model A	2.70	-5.59	11.00	0.523
ACO Model B	1.99	-5.49	9.46	0.602

Abbreviations: ACO is accountable care organization. DID is difference-in-difference. SMM is severe maternal morbidity.

[a] Medicaid ACO Model A represents the "Health system/managed care organization partnership" model type and ACO Model B represents the "Primary Care" model type.

[b] Difference-in-difference results are adjusted for age, a vector of diagnoses, number of insurance enrollment days during pregnancy, multiple gestation (if applicable), parity number, patient zip code characteristics, and include fixed effects for delivery month, county, and delivery hospital.

[c] Coefficients shown represent the difference-in-differences (DID) between Medicaid ACO Model A vs Model B vs Medicaid non-ACO before (q12016 – q42017) versus after (q32018 – q42020) Medicaid ACO implementation. All DID coefficients are reported as percentage points.

[d] SMM excludes blood transfusion and ventilation.

[e] Deliveries occurring in 2020 quarter 4 are excluded from postpartum measures.

© 2025 Cole MB et al. *JAMA Network Open*.

eTable 7-B. Association between Medicaid ACO model type[a] and perinatal care utilization when using an alternative, privately insured control group: difference-in-differences results[b] (2016-2020)

	Coefficient (DID - IRR)[c]	95% CI		P-value
Office visits – prenatal period, number				
non-ACO (private)	ref	ref	ref	ref
ACO Model A	1.00	0.99	1.02	0.603
ACO Model B	1.14	1.13	1.16	<0.001
Office visits – 60 days postpartum[d], number				
non-ACO (private)	ref	ref	ref	ref
ACO Model A	1.01	0.98	1.04	0.705
ACO Model B	1.14	1.10	1.17	<0.001
Office visits – 6 months postpartum[d], number				
non-ACO (private)	ref	ref	ref	ref
ACO Model A	0.98	0.95	1.01	0.125
ACO Model B	1.06	1.04	1.09	<0.001
ED visits – prenatal period, number				
non-ACO (private)	ref	ref	ref	ref
ACO Model A	0.98	0.91	1.05	0.581
ACO Model B	1.12	1.05	1.19	0.001
ED visits – 60 days postpartum[d], number				
non-ACO (private)	ref	ref	ref	ref
ACO Model A	0.95	0.83	1.09	0.447
ACO Model B	0.96	0.85	1.10	0.581
ED visits – 6 months postpartum[d], number				
non-ACO (private)	ref	ref	ref	ref
ACO Model A	0.98	0.88	1.09	0.689
ACO Model B	1.01	0.91	1.11	0.892

Abbreviations: ACO is accountable care organization. DID is difference-in-difference. SMM is severe maternal morbidity. ED is emergency department. IRR is incidence rate ratio.

[a] Medicaid ACO Model A represents the "Health system/managed care organization partnership" model type and ACO Model B represents the "Primary Care" model type.

[b] Difference-in-difference results are adjusted for age, a vector of diagnoses, number of insurance enrollment days during pregnancy, multiple gestation (if applicable), parity number, patient zip code characteristics, and include fixed effects for delivery month, county, and delivery hospital.

[c] Coefficients shown represent the difference-in-differences (DID) between Medicaid ACO Model A vs Model B vs Medicaid non-ACO before (q12016 – q42017) versus after (q32018 – q42020) Medicaid ACO implementation. All DID coefficients are reported as Incident Rate Ratios (IRRs), where IRR>1.0 indicates a positive association between the Medicaid ACO and the outcome.

[d] Deliveries occurring in 2020 quarter 4 are excluded from postpartum measures and deliveries occurring in 2020 quarters 3-4 are excluded from 6 month postpartum measures.

© 2025 Cole MB et al. *JAMA Network Open*.

Assessing pre-period trends in outcomes. As shown in eTable 8-A and 8-B below, we statistically assess whether there are differential trends in our outcomes between the treatment vs comparison groups within the pre-period. While we do not find any statistically significant pre-period differences (p<0.05 for all outcomes), we recognize that confidence intervals may be wide in some quarters, particularly for the non-ACO group, which has a smaller sample size than the treatment groups. Visual assessment of the study outcomes suggests that, while there is sometimes quarter-to-quarter noise (i.e. large standard errors), for the outcomes that we find to be statistically significant in our main analysis, there is also a relative improvement in these outcomes when qualitatively assessing the graphs. For instance, when assessing number of prenatal visits (Figure 1, main paper), Model B experiences a clear shift upwards in the post-period, whereas Model A experiences a shift downward (the non-ACO group also experiences a shift downward, but to a lesser extent than Model A). Of note, we adjust each figure's Y axis to have a limited range of values, in order to more clearly demonstrate changes; as a result, the data visually appears more "noisy" than it would if the Y axis leveraged a wider range.

eTable 8. Preperiod Trends for Medicaid ACO vs Medicaid non-ACO Deliveries, 2016-2017

eTable 8-A. Pre-period trends for Medicaid ACO model type[a] vs Medicaid non-ACO deliveries (2016-2017)

	Coefficient (DID - probability)[b]	95% CI		P-value
SMM during delivery[c] (per 10,000 deliveries)				
non-ACO	ref	ref	ref	ref
ACO Model A	1.41	-3.98	6.81	0.607
ACO Model B	3.06	-2.41	8.53	0.273
Cesarean section delivery, %				
non-ACO	ref	ref	ref	ref
ACO Model A	-0.38	-1.16	0.39	0.334
ACO Model B	-0.44	-1.21	0.33	0.263
Pre-term birth (<37 weeks), %				
non-ACO	ref	ref	ref	ref
ACO Model A	0.14	-0.33	0.60	0.569
ACO Model B	-0.18	-0.64	0.29	0.459
Timely postpartum visit, %				
non-ACO	ref	ref	ref	ref
ACO Model A	0.00	-0.68	0.67	0.989
ACO Model B	-0.21	-0.51	0.08	0.161
Postpartum depression screening[d], %				
non-ACO	ref	ref	ref	ref
ACO Model A	0.01	0.00	0.01	0.064
ACO Model B	0.01	-0.01	0.02	0.506
Postpartum glucose screening[d], %				
non-ACO	ref	ref	ref	ref
ACO Model A	-0.76	-3.75	2.23	0.619
ACO Model B	-0.26	-2.82	2.29	0.839

© 2025 Cole MB et al. *JAMA Network Open*.

Abbreviations: ACO is accountable care organization. DID is difference-in-difference. SMM is severe maternal morbidity.
[a] Medicaid ACO Model A represents the "Health system/managed care organization partnership" model type and ACO Model B represents the "Primary Care" model type.
[b] Coefficients shown represent the difference-in-differences (DID) between Medicaid ACO Model A vs Model B vs Medicaid non-ACO before (q12016) versus after (q42017). All DID coefficients are reported as percentage points.
[c] SMM excludes blood transfusion and ventilation.

© 2025 Cole MB et al. *JAMA Network Open*.

eTable 8-B. Pre-period trends for Medicaid ACO model type[a] vs Medicaid non-ACO deliveries (2016-2017)

	Coefficient (DID - IRR)[b]	95% CI		P-value
Office visits – prenatal period, number				
non-ACO	ref	ref	ref	ref
ACO Model A	1.00	0.99	1.01	0.882
ACO Model B	1.00	1.00	1.01	0.297
Office visits – 60 days postpartum, number				
non-ACO	ref	ref	ref	ref
ACO Model A	0.99	0.97	1.00	0.168
ACO Model B	0.99	0.98	1.01	0.303
Office visits – 6 months postpartum, number				
non-ACO	ref	ref	ref	ref
ACO Model A	1.00	0.99	1.01	0.786
ACO Model B	1.00	0.99	1.01	0.751
ED visits – prenatal period, number				
non-ACO	ref	ref	ref	ref
ACO Model A	1.01	0.98	1.03	0.616
ACO Model B	1.01	0.99	1.04	0.355
ED visits – 60 days postpartum, number				
non-ACO	ref	ref	ref	ref
ACO Model A	0.97	0.92	1.02	0.180
ACO Model B	0.97	0.92	1.02	0.223
ED visits – 6 months postpartum, number				
non-ACO	ref	ref	ref	ref
ACO Model A	1.00	0.96	1.04	0.945
ACO Model B	0.98	0.95	1.02	0.292

Abbreviations: ACO is accountable care organization. DID is difference-in-difference. SMM is severe maternal morbidity. ED is emergency department. IRR is incidence rate ratio.

[a] Medicaid ACO Model A represents the "Health system/managed care organization partnership" model type and ACO Model B represents the "Primary Care" model type.

[b] Coefficients shown represent the difference-in-differences (DID) between Medicaid ACO Model A vs Model B vs Medicaid non-ACO before (q12016) versus after (q42017). All DID coefficients are reported as Incident Rate Ratios (IRRs), where IRR>1.0 indicates a positive association between the Medicaid ACO and the outcome.

© 2025 Cole MB et al. *JAMA Network Open*.

eTable 8-C. Pre-period trends for Medicaid ACO model type vs Privately insured non-ACO deliveries (2016-2017) – for outcomes that were otherwise significant in main analyses

	Coefficient (DID - probability)	95% CI		P-value
Cesarean section delivery				
Medicaid non-ACO	ref	ref	ref	ref
Medicaid ACO Model A	0.0037	-0.0010	0.0083	0.121
Medicaid ACO Model B	-0.0007	-0.0051	0.0036	0.741
Timely postpartum visit				
Medicaid non-ACO	ref	ref	ref	ref
Medicaid ACO Model A	0.0035	-0.0006	0.0076	0.094
Medicaid ACO Model B	0.0023	-0.0015	0.0061	0.240
Postpartum depression screening[a]				
Medicaid non-ACO	ref	ref	ref	ref
Medicaid ACO Model A	0.0083	0.0047	0.0119	0.000
Medicaid ACO Model B	0.0027	-0.0005	0.0060	0.102
Office visits, prenatal				
Medicaid non-ACO	ref	ref	ref	ref
Medicaid ACO Model A	-0.1192	-0.2776	0.0392	0.140
Medicaid ACO Model B	-0.0125	-0.1550	0.1300	0.864
Office visits, 60 days PP				
Medicaid non-ACO	ref	ref	ref	ref
Medicaid ACO Model A	0.0052	-0.0102	0.0207	0.506
Medicaid ACO Model B	0.0085	-0.0058	0.0228	0.245
Office visits, 6 months PP				
Medicaid non-ACO	ref	ref	ref	ref
Medicaid ACO Model A	-0.0019	-0.0298	0.0260	0.895
Medicaid ACO Model B	-0.0211	-0.0469	0.0048	0.110
ED visits, prenatal				
Medicaid non-ACO	ref	ref	ref	ref
Medicaid ACO Model A	0.0059	-0.0074	0.0191	0.385
Medicaid ACO Model B	0.0108	-0.0017	0.0233	0.089

[a] Pre-period trend is statistically significant ($p<0.05$). Therefore, we are unable to draw valid conclusions about this outcome within the context of this sensitivity analysis.

© 2025 Cole MB et al. *JAMA Network Open*.

eTable 9. Difference-in-Differences Model Type Results Excluding 2020 Data

eTable 9-A. Difference-in-differences model type[a] results[b] excluding 2020 data

	Coefficient (DID - probability)[c]	95% CI		P-value
SMM during delivery[d] (per 10,000 deliveries)				
non-ACO	ref	ref	ref	ref
ACO Model A	-7.80	-53.99	38.38	0.741
ACO Model B	7.45	-38.33	53.23	0.750
Cesarean section delivery, %				
non-ACO	ref	ref	ref	ref
ACO Model A	-2.93	-5.73	-0.14	0.040
ACO Model B	-2.94	-5.69	-0.18	0.037
Pre-term birth (<37 weeks), %				
non-ACO	ref	ref	ref	ref
ACO Model A	-0.39	-2.10	1.32	0.658
ACO Model B	-0.24	-1.93	1.45	0.783
Timely postpartum visit, %				
non-ACO	ref	ref	ref	ref
ACO Model A	5.28	2.91	7.66	<0.001
ACO Model B	0.78	-1.57	3.14	0.515
Postpartum depression screening, %				
non-ACO	ref	ref	ref	ref
ACO Model A	5.31	3.18	7.45	<0.001
ACO Model B	8.16	6.07	10.25	<0.001
Postpartum glucose screening, %				
non-ACO	ref	ref	ref	ref
ACO Model A	-8.75	-18.42	0.92	0.076
ACO Model B	-5.53	-14.46	3.40	0.225

Abbreviations: ACO is accountable care organization. DID is difference-in-difference. SMM is severe maternal morbidity.

[a] Medicaid ACO Model A represents the "Health system/managed care organization partnership" model type and ACO Model B represents the "Primary Care" model type.

[b] Difference-in-difference results are adjusted for age, a vector of diagnoses, number of insurance enrollment days during pregnancy, multiple gestation (if applicable), parity number, patient zip code characteristics, and include fixed effects for delivery month, county, and delivery hospital.

[c] Coefficients shown represent the difference-in-differences (DID) between Medicaid ACO Model A vs Model B vs Medicaid non-ACO before (q12016 – q42017) versus after (q32018 – q42019) Medicaid ACO implementation. All DID coefficients are reported as percentage points.

[d] SMM excludes blood transfusion and ventilation.

© 2025 Cole MB et al. *JAMA Network Open*.

eTable 9-B. Difference-in-differences model type[a] results[b] excluding 2020 data

	Coefficient (DID - IRR)[c]	95% CI		P-value
Office visits – prenatal period, number				
non-ACO	ref	ref	ref	ref
ACO Model A	0.97	0.94	1.00	0.034
ACO Model B	1.09	1.06	1.12	<0.001
Office visits – 60 days postpartum, number				
non-ACO	ref	ref	ref	ref
ACO Model A	1.00	0.94	1.05	0.907
ACO Model B	1.11	1.05	1.18	<0.001
Office visits – 6 months postpartum, number				
non-ACO	ref	ref	ref	ref
ACO Model A	1.00	0.96	1.05	0.906
ACO Model B	1.07	1.02	1.12	0.008
ED visits – prenatal period, number				
non-ACO	ref	ref	ref	ref
ACO Model A	0.93	0.85	1.01	0.087
ACO Model B	1.06	0.97	1.15	0.194
ED visits – 60 days postpartum, number				
non-ACO	ref	ref	ref	ref
ACO Model A	0.94	0.78	1.13	0.490
ACO Model B	0.96	0.80	1.14	0.629
ED visits – 6 months postpartum, number				
non-ACO	ref	ref	ref	ref
ACO Model A	0.98	0.85	1.11	0.711
ACO Model B	0.99	0.87	1.13	0.931

Abbreviations: ACO is accountable care organization. DID is difference-in-difference. SMM is severe maternal morbidity. ED is emergency department. IRR is incidence rate ratio.

[a] Medicaid ACO Model A represents the "Health system/managed care organization partnership" model type and ACO Model B represents the "Primary Care" model type.

[b] Difference-in-difference results are adjusted for age, a vector of diagnoses, number of insurance enrollment days during pregnancy, multiple gestation (if applicable), parity number, patient zip code characteristics, and include fixed effects for delivery month, county, and delivery hospital.

[c] Coefficients shown represent the difference-in-differences (DID) between Medicaid ACO Model A vs Model B vs Medicaid non-ACO before (q12016 – q42017) versus after (q32018 – q42019) Medicaid ACO implementation. All DID coefficients are reported as Incident Rate Ratios (IRRs), where IRR>1.0 indicates a positive association between the Medicaid ACO and the outcome.

© 2025 Cole MB et al. *JAMA Network Open*.
