# Supplementary material for: Medicaid Accountable Care Model Designs and Maternal Health Measures
Source: JAMA Netw Open. 2025 Oct 8;8(10):e2536565. doi: 10.1001/jamanetworkopen.2025.36565 (PMC12509007; doi:10.1001/jamanetworkopen.2025.36565)
Supplement: Supplement 2. — Data Sharing Statement [file jamanetwopen-e2536565-s002.pdf]

Data Sharing Statement

Cole. Medicaid Accountable Care Model Designs and Maternal Health Measures. *JAMA Netw Open*. Published October 08, 2025. doi:10.1001/jamanetworkopen.2025.36565

Data
Data available: No

Additional Information
Explanation for why data not available: These data are governed by a data use agreement (DUA) that does not allow for data sharing, as they are claims data.
